# Clinical use of a modified release methylphenidate in the treatment of childhood attention deficit hyperactivity disorder

**DOI:** 10.1186/1744-859X-10-25

**Published:** 2011-09-30

**Authors:** Inyang Takon

**Affiliations:** 1 QE II Hospital, East and North Hertfordshire NHS Trust, Welwyn Garden City, UK

## Abstract

Attention deficit hyperactivity disorder (ADHD) is the most commonly diagnosed neurobehavioural disorder in childhood, affecting over 5% of children worldwide. As well as the core symptoms of inattention, hyperactivity and impulsivity, patients often exhibit learning difficulties and impairment in social functioning. The frequency of referral is higher for boys than for girls (about 2:1), and girls are generally older at the time of referral.

Pharmacological therapy is considered the first-line treatment for patients with severe ADHD and severe impairment. Stimulant medications are licensed in the UK for the management of ADHD in school-age children and young people, and are effective in controlling ADHD symptoms.

While immediate-release preparations of methylphenidate (MPH) have proven effective in the treatment of ADHD, there are a number of problems associated with their use, most notably compliance, stigma and medication diversion. Modified release preparations are now available that overcome the need for multiple daily dosing, and which offer different MPH release profiles, thereby enabling the physician to match the medication to the patient's particular requirements.

This review describes the diagnosis, referral and treatment pathways for patients with ADHD in the UK and the practical considerations when initiating pharmacological treatment. The clinical experience of treating ADHD with a modified-release MPH preparation (Equasym XL^®^) is illustrated with case studies.

## Introduction

Attention deficit hyperactivity disorder (ADHD) is the most commonly diagnosed neurobehavioural disorder in childhood; a recent meta-regression analysis estimated a worldwide prevalence of 5.29% among children and adolescents [1]. It is characterised by inappropriate levels of inattention, hyperactivity and impulsivity, and often accompanied by comorbid symptoms such as aggressive behaviour, depressive mood, anxiety and tics [2]. Furthermore, learning difficulties [3] and impairment in social functioning [4] are frequently observed in patients with ADHD. The symptoms of ADHD decline with increasing age, and although most patients with ADHD no longer meet the full criteria for the disorder when they reach adulthood, up to 50% of childhood cases continue to show clinically relevant symptoms during adolescence and adult life [5].

In the UK, patients with suspected ADHD, identified through concerns from school (teachers, special educational needs coordinators and educational psychologists) or family members, are brought to the attention of the general practitioner (GP) via parents. Children are then referred for further specialist assessment for ADHD. The referral pathway varies in different parts of the country, with paediatricians carrying out the diagnostic assessment in some areas and child psychiatrists in others; some centres provide a joint service involving both paediatricians and child psychiatrists.

Children referred to their GP usually present with concerns of inattention, hyperactivity and impulsivity. Symptoms impact on their academic achievements and social interaction at school; home life can also be very difficult, as children with ADHD are constantly active and demanding, which tends to cause conflicts with their families. The frequency of referral is higher for boys than for girls (about 2:1), and girls are generally older at the time of referral. This is probably because girls are more likely to have the predominantly inattentive subtype of ADHD [6], which, because the symptoms are less striking, is often identified only when poor academic performance is noticed. Symptoms also vary with age and may be less obvious, but equally impairing, in older adolescents. Notable risk factors for developing ADHD include family history of ADHD [7], preterm birth and smoking or drinking during pregnancy [8].

Pharmacological therapy is considered the first-line treatment for patients with severe ADHD and severe impairment [9]. Stimulant medications, such as methylphenidate (MPH) and dexamfetamine, and the non-stimulant, atomoxetine, are licensed in the UK for the management of ADHD in school-age children and young people, and are effective in controlling ADHD symptoms [9]. The guidelines produced by the UK National Institute of Clinical Excellence (NICE) [9] recommend MPH as the first-choice medication for patients without comorbidities, for those with comorbid conduct disorder and when tics, Tourette's syndrome, anxiety disorder and stimulant misuse or risk of stimulant misuse are present; however, MPH should be used with caution in such patients. Although the mechanism of action is not completely understood, MPH is known to be associated with an increase in dopamine and norepinephrine levels in the extraneuronal space, probably owing to reuptake inhibition. This is thought to improve neurotransmission and to mediate the beneficial effects of MPH on ADHD symptoms [10]. MPH is primarily metabolised by de-esterification to ritalinic acid, which is pharmacologically inactive; therefore, MPH has a low absolute bioavailability, and a short half life (2-3 h) and duration of action [11-13].

Immediate-release formulations of MPH (MPH-IR) have been used since the 1960s to control ADHD symptoms. With MPH-IR, peak plasma concentrations and optimum behavioural effects are reached approximately 1.5-2.0 h after administration [14]. The limited duration of action means that most children require two or three daily doses to maintain control of ADHD symptoms throughout the day [15]. However, medication doses given during the school day can cause compliance issues and problems related to privacy, stigmatisation by classmates, accountability of the school administration and potential abuse or diversion due to availability of the medication in school [10,16]. To eliminate the need for multiple daily doses, modified-release MPH (MPH-MR) formulations have been designed which combine IR and delayed-release (DR) components, and produce a rapid onset of therapeutic effect while having a sufficient duration to eliminate the need for additional dosing. MR formulations avoid the oscillations in plasma concentration seen with multiple dosing of MPH-IR, but at the same time present a biphasic profile rather than a flat profile, preventing the development of acute tolerance [17]. MPH-MR formulations approved in the UK are listed in Table 1. The pharmacokinetic (PK) profile over time of these formulations is distinct, and their effect on behaviour parallels the blood concentration of MPH. MPH-MR 50:50 (Medikinet XL^®^) and MPH-MR 30:70 (Equasym XL^®^) have been shown to be equivalent to twice-daily MPH-IR [18,19], while MPH-OROS (Concerta XL^®^) is equivalent to MPH-IR, three times daily, although at a 20% higher dose [20]. Several comparative studies between MPH-MR products have shown the relative efficacies over different periods of the day, depending on the release profile [21-23]. Having different MPH-MR proportions with differing release profiles allows tailoring of treatment to each patient, depending on the desired profile of symptom control throughout the day. This review focuses on the practical and clinical experience with the MR preparation MPH-MR 30:70 in a UK paediatric ADHD clinic.

**Table 1:** Extended-release preparations of methylphenidate currently available in the UK

Brand name	UK marketing company	Technology	IR:DR ratio
Medikinet XL	Flynn Pharma Ltd	Multiparticulate bead system	50:50
Equasym XL	Shire Pharmaceuticals Ltd	Multiparticulate bead system	30:70
Concerta XL	Janssen-Cilag Ltd	Osmotic release oral system	22:78

DR = delayed release; IR = immediate release.

### Practical considerations for initiating MPH treatment

At the start of treatment with any MPH formulation, careful dose titration is necessary. IR and long-acting formulations of MPH can both be used to initiate treatment of ADHD [24-26]. In some cases, IR preparations are slowly titrated by weekly increments of 5-10 mg/day according to tolerability and the degree of efficacy observed, up to a maximum daily dose of 60 mg; IR preparations are taken in divided doses, usually at breakfast and lunch [24]. Some patients are stabilised on MPH-IR and then switched to a corresponding dose of MPH-MR. However, it is commonly accepted practice now in most areas of the UK to initiate ADHD therapy directly with the lowest available dose of MPH-MR when this medication has been chosen as the treatment of choice. The dose should be incremented slowly, every 1-2 weeks, until an effective dose is reached. Children as young as 6 years old can have treatment initiated with the capsule formulations of MPH-MR, as these can be opened and the contents given in soft food [24,26].

From a clinical perspective, the regimen that achieves satisfactory symptom control at the lowest total daily dose and with the fewest adverse events (AEs) should be employed. In cases where treatment effects wear off too early, patients can be given an extra dose of MPH-IR in the late afternoon or early evening if needed [24,26]; for example, for doing homework or maintaining focus during after-school activities. However, caution should be applied in children with significant appetite suppression or sleep problems, as the additional MPH-IR may negatively affect them.

The choice between treatment options should be determined on an individual basis, with particular consideration of the requirement for symptom control in the latter part of the day. MPH treatment is generally well tolerated by patients and severe AEs are rare. Increased insomnia, other sleep-related problems and reduced appetite are common, and there is some evidence for increased levels of emotionality, social withdrawal, nausea and stomach aches, although it is not clear which of these AEs are related to pre-existing problems associated with ADHD prior to treatment [27]. Furthermore, treatment tolerability does not appear to be predictable on the basis of patients' clinical characteristics [27].

Long-term side effects of MPH include weight loss from reduced appetite and reduction in height velocity [28]; the magnitude of such effects has been controversial for many years [29]. The cause of the reduction in height velocity is not entirely clear [30]. In some cases, it is thought that it may be secondary to the effect of MPH on weight [30]. It has also been suggested that ADHD itself may be associated with temporary deficits in height gain during mid adolescence, supporting the hypothesis that this effect could be a consequence of the disease rather than its treatment [31]. The height of most children normalises with increased age and most achieve normal adult height; however, in a few cases where height is significantly affected during ongoing monitoring, children should be referred to a paediatric endocrinologist to rule out any undiagnosed growth problems.

### Equasym XL

This MPH-MR 30:70 formulation of MPH contains a 30:70 ratio of IR to DR MPH by weight [17]. It uses a multiparticulate bead delivery system in a capsule, in which each bead acts as a drug reservoir; a distinct advantage of this system is that capsules can be swallowed whole, or their contents can be sprinkled onto a small amount of apple sauce, without altering the bioavailability of the MPH [32]. This may be helpful in very small children or those with swallowing or gastrointestinal issues.

The efficacy of MPH-MR 30:70 in children with ADHD has been demonstrated in three 3-week randomised, double-blind studies: a placebo-controlled non-inferiority study compared with a twice-daily IR formulation [19] and a parallel-group, placebo-controlled clinical trial [33], both conducted in a community setting, and a comparative, laboratory classroom study, (Comparison Of Methylphenidates in an Analog Classroom Setting (COMACS)) [23], with several secondary analyses [27,34-37]. Study details are shown in Table 2.

**Table 2:** Clinical studies assessing the efficacy of MPH-MR 30:70

Parameter	Information
Study	Greenhill *et al*., 2002 [33]

Type	Community setting. Multicentre, placebo-controlled, USA.

Population characteristics	6-16 years old, mild ADHD

Interventions	Placebo (163), MPH-MR 30:70 (158)

Dose and duration	20-60 mg/day, titrated to optimum efficacy (3 weeks)

Outcome measures	Teacher and Parent Conners' Global Index, CGI-S, CGI-I

Conclusions	Equasym XL administered once daily in the morning was well tolerated and significantly more effective than placebo in controlling ADHD symptoms throughout the school day. Symptom control was achieved in the morning and afternoon.

Study	Findling *et al*., 2006 [19]

Type	Community setting. Non-inferiority, Australia, Canada and USA.

Population characteristics	6-12 years old, ADHD

Interventions	Placebo, Ritalin (IR), MPH-MR 30:70

Dose and duration	According to prestudy MPH regimen. Equasym XL, 20-60 mg/day; Ritalin, 10-30 mg twice daily (3 weeks).

Outcome measures	Teacher's and parent's IOWA Conners' Rating Scale

Conclusions	Equasym XL once daily was statistically non-inferior to Ritalin twice daily in the treatment of school-age children with methylphenidate-responsive ADHD. Both Equasym XL and Ritalin were superior to placebo in controlling ADHD symptoms, and were well tolerated.

Study	Swanson *et al*., 2004 [23]

Type	Laboratory-classroom setting. Double-blind, three-way, crossover study, USA.

Population characteristics	6-12 years old, confirmed ADHD

Interventions	MPH-MR 30:70, Concerta XL

Dose and duration	Assigned to low (20 or 18 mg/day), medium (40 or 36 mg/day) or high (60 or 54 mg/day) dose according to prestudy MPH dose. Crossover design, so all patients received both active agents and placebo for 7 days each throughout a 3-week period.

Outcome measures	SKAMP Rating Scale, PERMP

Conclusions	There were statistically significant differences between the efficacy of Equasym XL and Concerta in children with ADHD in the laboratory school setting. As predicted by the PK/PD model, clinical superiority at any time point was achieved by the formulation with the highest expected plasma methylphenidate concentration.

ADHD = attention deficit hyperactivity disorder; CGI-I = Clinical Global Impression-Improvement; CGI-S = Clinical Global Impression-Severity; IOWA = inattention/overactivity with aggression; IR = immediate release; PERMP = Permanent Product Measure of Performance; SKAMP = Swanson, Kotkin, Agler, M-Flynn and Pelham.

These trials show that MPH-MR 30:70 is an effective and well tolerated once-daily treatment for ADHD [19,27,33-37]. However, the COMACS secondary analyses also made it clear that different patients respond in different ways to MPH-MR formulations [35,36]. This heterogeneity of response is an important consideration when deciding treatment options for children with ADHD. COMACS also showed that parents and teachers assess symptoms differently, highlighting the need for comprehensive assessment measures [37]. It should also be noted that clinical trial data represent the average values from variable populations. Therefore, for each individual, the most rational initial choice (based on clinical trial results) might not be the best treatment. Reliable, cost-effective, predictive factors that can be used in everyday clinical practice are needed.

### Challenges in clinical practice

In the specialist ADHD clinic in East and North Hertfordshire, UK, assessment is by use of the P4 screening questionnaire, which is a 33-item screening questionnaire based on the *Diagnostic and Statistical Manual, fourth edition *(DSM-IV) criteria and which assesses the core symptoms of ADHD: concentration, hyperactivity and impulsivity. The P4 was designed by one of the consultant child psychiatrists in the team and has been in use for 15 years as one of the tools available for screening the core symptoms of ADHD. The questionnaire has shown good correlation with information received from school and home in identifying children with ADHD, although it has not been validated in population studies. The diagnostic assessment in our unit involves: (1) diagnostic interview with the parent/carer and the child/young person in the clinic (the interview lasts on average 1 h). (2) School observation of the primary school child to assess for core symptoms of ADHD. This observation is usually carried out by the ADHD nurse specialist, using a structured form. The process includes observation of the child in the playground to observe social interaction with peers, observation of the child during lessons to check for impulsive and inattentive behaviours as well as overactivity, and a review of the child's completed class work. (3) Review of the screening questionnaires (P4) sent to home and school. Screening questionnaires are usually sent to the different subject teachers for the secondary school child.

After the conclusion of the diagnostic assessment by the paediatrician, the child psychiatrist, or both, a discussion is held with the parents and the young patient about the diagnosis and the different types of management options available. Information on where to access further parenting courses in managing the child's behaviour and psychoeducational materials are made available. When pharmacological therapy is recommended, the various types of medication available and their risks and benefits are discussed, and it is general practice to make this as visual as possible for the child and his/her parents by showing them dummy tablets of the proposed medication. Each of the child's difficulties is considered, to evaluate areas of particular concern. It is worth noting that the majority of parents accept pharmacological treatment for their children when indicated, although it is worth spending some time explaining how the medication works and allowing them to ask any questions they might have. Families also have further access to the ADHD nurse who sees the families after the initial clinic appointment and who discusses further questions that families may have and provides information on additional resources needed. Consent is usually obtained from the families to contact the school, and information on managing ADHD within the school is also forwarded to the class teacher.

In primary school children, who carry out most of their schoolwork in school with little requirement for homework after school, preparations such as MPH-MR 30:70 are very useful. The child feels better as all the medication is taken at home before leaving for school and there is a lower risk of poor compliance, as parents ensure the child takes the medication. In addition, there is a lower risk of stigmatisation in school.

The ADHD nurse specialist is a key team member in the East and North Hertfordshire ADHD service. This nurse will usually follow-up, by telephone, with the family, 1-2 weeks after the medication regimen has started in order to assess the child's response to the medication, and to start titration to the therapeutic dose. All follow-up visits use a structured *pro forma *that is used to review the core symptoms of ADHD and side effects of medication (in children treated with medication). The instrument also captures information on progress with learning and any emotional concerns. A summary of discussions with the family is maintained, including an action plan and plan for reviews.

Children started on medication are usually seen in the clinic after 1 month, and the optimal dose is determined. They are subsequently followed up on a 6-monthly basis if their symptoms are stable, and their height, weight and blood pressure are monitored; this is sometimes performed by the ADHD nurse specialist in the nurse-led clinic. Parents are instructed to contact the ADHD team (nurse specialist or clinician) if they have any concerns that require an earlier review. Side effects are usually transient and tend to subside. Children treated for ADHD with more complex issues, who have poor tolerance to medication or who experience side effects are usually seen more frequently, and follow-up varies from monthly to 3-monthly. There is flexibility within the service to see children earlier if the need arises. The structured *pro forma *described above is used during all follow-up visits, including telephone consultations.

It is useful to have an up-to-date progress report from the school for the follow-up visits. This helps to monitor progress on the core symptoms of ADHD and also to find out about school, home and sibling progress. Where there is a lack of response to the medication, it is advisable to explore possible reasons through a discussion with the child, rather than stop treatment abruptly.

Children who are stable on the 8-h preparation of MPH-MR 30:70 can be prescribed an additional MPH-IR dose in the evening when they make the transition to secondary school. This prevents changing the preparation to an entirely different one, particularly when it has been well tolerated, and extends symptom control to allow management of homework or other after-school activities.

### Managing side effects

The majority of children respond well to long-acting MPH, although a few patients show appetite suppression and other side effects such as emotional concerns, anxiety, mood changes and tics. In some cases, temporary withdrawal of long-acting MPH may be necessary to see if the side effects subside.

To manage poor weight gain, children may receive additional calories in the form of milk shakes or additional foods recommended by dieticians. With the 8-h MPH preparation there is usually a catch-up period in the evenings, when the medication effect wears off and children eat normally. Children are usually advised to eat a substantial breakfast before taking their medication and most are able to eat a healthy portion of food in the evening.

Children with reduced height velocity may need to discontinue the medication temporarily if their height velocity continues to drop. As mentioned above, referral to a paediatric endocrinologist will determine whether there is an underlying growth problem.

Headaches and abdominal pain usually improve after the first week of treatment; however, if they persist, analgesia can be given to relieve the symptoms. Emotional lability also usually improves with time; if it persists, the dose of the medication should be reviewed.

### Illustrative case studies of patient-tailored treatment with MPH-MR 30:70

#### Case study 1

Patient 1, age 14 years, presented with behavioural difficulties at home and school, and was first seen by clinicians following referral to the ADHD clinic at the age of 10 years. Concerns raised included challenging behaviour, impulsive behaviour, hyperactivity and fidgeting. Patient 1 lashed out very easily and struggled to calm down when he had an outburst; he had many outbursts at school, which made his peers wary of him, and was worried about his social skills.

Patient 1 had some initial risk factors. He was born following a full-term pregnancy, weighed 3.5 kg and was in the special care baby unit for 2 days as a result of low blood sugar. He was the second child born to his mother. There was a history of domestic violence from patient 1's father, and his mother also had a history of psychiatric illness. Patient 1 had normal motor development milestones, but his speech and language development were delayed, and he also suffered from sleep difficulties.

Patient 1 was diagnosed with ADHD at age 6.5 years in a different county but had not been treated with medication. Patient 1 and his mother then relocated to a different county and a further review of his symptoms was requested following increasing concerns regarding his behaviour at home and at school. The assessments consisted of a diagnostic interview by clinicians and a screening of the information received from home and the school. It was confirmed that patient 1 had ADHD combined type.

Patient 1 started treatment with MPH-OROS, but significant symptom control was not achieved; his dosage was steadily titrated up to the maximum recommended dose of MPH-OROS (72 mg) but he felt that the medication effect wore off easily and also affected his appetite. Patient 1's medication was subsequently changed to 50 mg/day MPH-MR 30:70, and then titrated up to 60 mg/day, which he tolerated better than MPH-OROS. He made significant improvements: his concentration and his school grades improved (he also moved to a smaller school). Patient 1's weight, height and blood pressure were normal for his age.

Patient 1 is now making good progress in school and is generally happier, as confirmed by school reports, parental reports of school progress and by the medication monitoring document. He has also been able to form friendships. He is being followed up every 6 months in the ADHD clinic.

#### Case study 2

Patient 2 was referred at age 5.5 years, following review in a child development centre, for concerns relating to poor concentration, impulsive behaviour and poor interaction in school. Patient 2's difficulties first became apparent when she was in preschool, where she required one-to-one support. She had problems with balance and motor skills, and walked with her feet turned in. She also had sleep difficulties and was very restless during sleep. At nursery, she had been functioning at an average level. Patient 2's behaviour had a significant impact on the whole family; she had defiant and attention-seeking behaviours.

Patient 2 was born 1 week after her due date of birth; there were no concerns following her birth and she had been in good health. There was a family history of psychiatric illness in her mother's grandfather, as well as a history of hyperactivity and defiant behaviours in patient 2's cousin. Patient 2's clinical examination showed joint laxity but her neurological examination was normal.

Patient 2 had a diagnostic assessment for ADHD and was found to have very high scores for inattention, hyperactivity and impulsivity, leading to a diagnosis of ADHD combined type with comorbid oppositional defiant disorder. ADHD management was discussed with the family, and her parents' decision was to try behavioural strategies at home and at school, and review patient 2's behaviour and progress in 1 year.

At 1 year later, her sleep problems had worsened; she was started on melatonin to help with her sleep, which improved slightly, but continued to have significant ADHD symptoms that had an impact on her learning. Patient 2 was started on MPH-MR 30:70 10 mg to help control ADHD symptoms during the school day and her dose was gradually titrated to 30 mg once daily. Patient 2's ADHD and comorbid symptoms improved, including her concentration, her play and social skills, her relationship with friends, parents and sibling, and her sleep difficulties. Her dyspraxia and motor difficulties also improved.

Patient 2 has made good progress on MPH-MR 30:70 30 mg and has had no side effects; she continues to have 2 mg of melatonin at night to improve her sleep pattern. She is being followed up on a 6-month basis.

#### Case study 3

Patient 3, age 10 years, was referred at age 8 years with significant concerns about her behaviour in school. She was very talkative in class, fidgety and disruptive.

Patient 3 was born following a normal pregnancy and had no problems after birth. She was an only child and was brought up following a very strict routine. Her developmental milestones were advanced, and she had many falls and emergency-room visits in the first few years of life. She also struggled significantly with learning and was found to have dyslexia. Although her dyslexia was well supported, she struggled continuously with her concentration; she frequently got into trouble in school and this caused significant difficulties for her parents, whose work life was constantly disrupted by calls from the school.

Patient 3's parents had her referred for further assessment for ADHD. The positive and problem profile questionnaire (similar to the strengths and difficulties questionnaire) was sent to her parents and the school, and school observation was arranged by the ADHD nurse specialist. Patient 3 showed borderline scores for all the core symptoms of ADHD.

Patient 3 was initially started on MPH-IR, 10 mg twice daily, following her parents' request for a short-acting preparation, to help them monitor her response during medication titration. The effect of the medication wore off quickly so her symptoms were not fully controlled. Her medication was then switched to MPH-MR 30:70, 30 mg once daily, to which she responded better. Patient 3 was satisfied with the once-daily dosage of MPH-MR 30:70 as she did not need to take medication in school and be different from her peers. Patient 3's relationship with her peers improved, and although she had slight appetite suppression whilst on the medication, she was able to have a normal evening meal with her family when she got back home, after the medication effect wore off.

Patient 3 has made good progress on MPH-MR 30:70 and her symptoms are well controlled during the school day. She is due to start secondary school education and the plan is for patient 3 to continue taking MPH-MR 30:70 for the school day, with the option to add an MPH-IR preparation for the late afternoon when she is required to concentrate on homework. Patient 3 is satisfied with continuing the medication in secondary school.

#### Case study 4

Patient 4, age 10 years, had a long history of poor concentration, and defiant and challenging behaviour. She was born following a normal pregnancy and delivery, and had a history of delay in all areas of her development; she walked late, talked late (age 3) and found mainstream schooling very challenging.

Patient 4's parents separated when she was 4 years old, and a shared contact arrangement with both parents was agreed. After the separation of her parents, patient 4's behaviour became increasingly challenging. She found it difficult to settle in mainstream school and made little progress in her learning; she was, therefore, transferred to a special school for children with moderate learning disabilities. Patient 4's behaviour continued to deteriorate in the special school. She was very impulsive, defiant and constantly fidgety; she lashed out frequently at teachers and other children, and had a very poor concentration span. She had a structured behavioural management plan in school as well as a period of monitoring. However, although her behaviour improved slightly, she made no progress in her learning, as reflected by her poor school grades and her teachers' reports, and she continued to have problems with concentration and constant fidgeting.

Patient 4 was assessed for ADHD and her symptoms met the diagnostic criteria for ADHD with comorbid oppositional defiant disorder. She was started on a low dose of MPH-IR and showed a slight improvement but had significant rebound symptoms and several emotional side effects. She was moody, very tearful and her behaviour was of concern at home and at school. Her medication was then changed to MPH-MR using MPH-MR 30:70. She showed a slight improvement for a few weeks at a dose of 20 mg/day but had significant problems with poor appetite and poor sleeping patterns; according to her parents, she was also more aggressive on MPH-MR 30:70. The dose was reduced to 10 mg without any appreciable reduction of side effects. As she did not tolerate even small doses of MPH, MPH-MR 30:70 was discontinued and clonidine (starting at 25 μg once daily for 1 week, titrated up to 25 μg twice daily for 1 month), a non-stimulant preparation, was started to help with ADHD symptoms. Although clonidine is not currently approved in ADHD, evidence suggests it may have some benefit [38]. After 1 month at 25 μg twice daily, clonidine was also discontinued as patient 4 still showed many side effects and appeared subdued, very moody and tearful on medication.

Patient 4 had all medication discontinued, and a period of monitoring was carried out with more behavioural support and one-to-one management. She continued to struggle with poor attention and concentration, showed a lack of progress with learning and exhibited high levels of anxiety. A meeting of health, education and social services professionals was held to address patient 4's needs. She was reassessed by the doctor and diagnosed with ADHD, comorbid oppositional defiant disorder and additional comorbidities, such as anxiety symptoms and learning difficulties. She was also assessed for autistic spectrum disorder but did not meet the diagnostic criteria.

Patient 4 was trialled on atomoxetine and given access to a nurse support service for patients on this medication, run by an independent provider. She was started on 10 mg once daily for 2 weeks, titrated to 18 mg once daily for 2 weeks, and then maintained on 25 mg once daily thereafter. Titration was performed slowly, with close monitoring. Patient 4 showed a significant response to atomoxetine: her core ADHD symptoms improved, she was less anxious and her appetite and sleep improved. She still has some residual symptoms of impulsivity but, overall, she has made progress through a combination of atomoxetine and behavioural strategies.

## Conclusions

The early identification of ADHD symptoms in children is essential to prevent the complications that arise from late diagnosis. Children with risk factors for ADHD (such as prematurity, low blood sugar, difficulties around the time of birth with hypoxic brain injuries or infections, and children who develop difficulties with concentration and learning) [39-41] need to be screened and assessed for ADHD. Sleep difficulties are also common in children with ADHD [42] and may be an early sign in some cases. Sleep problems in children who also have a diagnosis of ADHD may improve upon treatment with medication [43].

Treatment with MPH-MR 30:70 prevents disruption in the child's school day programme by removing the need for a lunchtime medication dose administered in school. Parents who have doubts about medication treatment and concerns about the child taking medication in school are more likely to accept treatment with a flexible once-a-day preparation such as MPH-MR 30:70.

Children treated with MPH-MR 30:70 can have problems with appetite suppression, but as the effect wears off in the evening, the child is usually able to eat a normal evening meal, which prevents weight loss in most children.

Some children with learning difficulties and ADHD can have a poorer response to MPH compared with children without comorbidities. In these cases, the side effects of MPH-MR 30:70 can be seen with very small doses of MPH. Children with poor response to MPH-MR 30:70 and other MPH preparations should have their symptoms re-evaluated to rule out comorbidities such as anxiety disorder and autistic spectrum disorder, which may account for the poor response.

Providing adequate support for children treated with MPH-MR 30:70 is essential to ensure compliance with treatment.

## Consent

Formal consent has been obtained by the Author for all cases reported in the article.

## Competing interests

Support was received from Shire Pharmaceuticals Ltd to attend conferences.

## Author information

IT is the lead paediatrician for ADHD services in East Hertfordshire, UK, where she runs a weekly joint ADHD clinic with the Child and Adolescent psychiatrist and works within an ADHD specialist team. IT also sees children with other neurodisability issues who may have comorbid ADHD, where the presentation may be more complex and challenging to manage. IT has vast experience in managing children with complex ADHD. She has 18 years of experience in paediatrics and also has extensive experience in the use of psychopharmacologic agents in managing children with ADHD.
